# Entrepreneurial Leadership and Employee Wellbeing During COVID-19 Crisis: A Dual Mechanism Perspective

**DOI:** 10.3389/fpsyg.2022.800584

**Published:** 2022-07-18

**Authors:** Muhammad Bilal, Shafaq Arif Chaudhry, Imran Sharif, Owais Shafique, Khurram Shahzad

**Affiliations:** ^1^Lahore Business School, The University of Lahore, Lahore, Pakistan; ^2^Department of Islamic and Conventional Banking, Institute of Business, Management and Administrative Sciences, Islamia University of Bahawalpur, Bahawalpur, Pakistan; ^3^Northern University, Nowshera, Pakistan

**Keywords:** entrepreneurial leadership, work uncertainty, proactive work behavior, psychological wellbeing, sustainable employability, COVID-19

## Abstract

This study examined the potential impacts of entrepreneurial leadership on followers' psychological wellbeing and proactive work behavior through sustainable employability and work uncertainty in a sample of 218 employees employed in SMEs of Pakistan. Hierarchical regression results demonstrated that entrepreneurial leadership was positively connected with sustainable employability and negatively linked with work uncertainty. Sequentially, sustainable employability was positively correlated with proactive work behavior and employees' psychological wellbeing, and work uncertainty was negatively associated with proactive work behavior and employees' psychological wellbeing during the COVID-19 crisis. Furthermore, bootstrapping confirmed the mediation effects of work uncertainty and sustainable employability on proactive work behavior and the psychological wellbeing of employees. Sustainable employability did not mediate the relationship between entrepreneurial leadership and psychological wellbeing. Mediators, sustainable employability, and work uncertainty positively linked employees' psychological wellbeing and proactive work behavior. The results highlighted the significant roles of sustainable employability and work uncertainty and interpreted why entrepreneurial leadership may affect employees' positive behaviors.

## Introduction

The COVID-19 pandemic generated a persistent environment of uncertainty regarding health, social relations, finances, and economic conditions. Isolation, social distancing, and work uncertainty severely damage employees' psychological wellbeing, which further leads to a radical escalation in the states of mental stress and hopeless thoughts (Kawohl and Nordt, 2020). Furthermore, losing control and distressing social interaction leads to psychological disturbance, leading to economic, and work uncertainty (Reneflot and Evensen, 2014). Again, as more than 190 million individuals are unemployed, individual concern related to future employment and stress associated with the continuity of current work has been raised (Gangopadhyaya and Garrett, 2020). Such conditions have disturbed the sustainable employability and psychological wellbeing of employees. Considering this, researchers have recommended investigating the antecedent of sustainable employability (Fleuren et al., 2020).

Currently, we are experiencing turbulent environmental circumstances, unpredictable technological fluctuations, amplified competition, and integrated with the COVID-19 pandemic that appeared abruptly in the universe and its adverse upshot for the sustainability of all organizations. These situations force leaders to understand that they must inspire individuals to be proactive (Schmitt et al., 2016; Hu et al., 2018). Leaders can play a significant role in creating a caring milieu, for instance, by displaying ample backing for employees' struggles, authorizing them to incur additional charges by stimulating their autonomy (Avolio and Bass, 1995, p. 202). Furthermore, the research results regarding the leaders' supportive role in improving proactive behavior are varied. For example, some researchers stated an optimistic association between leader backing and different practices of proactive behavior (e.g., Axtell et al., 2000; Ramus and Steger, 2000; Madjar et al., 2002), and few showed the reverse (e.g., Oldham and Cummings, 1996; Frese et al., 1999; Parker et al., 2006). Nevertheless, the unpredictable results suggest the necessity to investigate intensely how leader support impacts employees' proactive behavior and wellbeing.

Entrepreneurial leaders envisage the business prospects, generate novel notions, and endeavor to increase firm's outcome (Fontana and Musa, 2017); such leaders hold exploration and exploitation of opportunity as prime objectives to encourage improvement in the firm performance (Koryak et al., 2015; Fontana and Musa, 2017). Entrepreneurial leadership is imperative for nurturing innovation and creativity, as proved by various studies (Cai et al., 2019). Leaders with entrepreneurial mindsets inspire their workers to engage in opportunity exploration and cultivate novel services, products, and business operation activities to achieve their objectives in the implementation process (Renko et al., 2015; Bagheri, 2017; Bagheri and Akbari, 2018). Thus, while chasing opportunity-oriented responsibilities, entrepreneurial leaders increase the individuals' inventive and initiative talent in ascertaining novel practices and ideas (Renko et al., 2015). All of which are compatible with the components of SDT (Deci and Ryan, 2013), which propose that individuals behave in a more positive way when their leader trusts them and gives them autonomy to perform their job. Entrepreneurial leaders also promote competence and relatedness by encouraging the exploration and exploitation of novel ideas to achieve the objectives (Renko et al., 2015; Bagheri, 2017). Entrepreneurial leaders' creative action and risk-taking behavior improve employees' self-reliance and competence to behave proactively by taking initiatives.

Self-determination theory is a comprehensive model of wellness, individual goals, and motivation (Ryan and Deci, 2017) and is appropriate for discussing workplace motivation and involvement in the contemporary era (Gagné et al., 2018). Self-determination theory offers a basic framework for individuals and how a situation such as work uncertainty or sustainable employability supports or spoils employees' wellbeing and motivation. In this connection, self-determination theory is adequate and efficient in aligning societal shift to individual autonomy, demonstrating a fact-base process of involvement and motivation with robustness to suspend conventional transaction reasoning about workplace motivation. Furthermore, self-determination theory has revealed how its motivational “act” envisage vital organizational up shots like wellbeing (Gagné and Deci, 2005; Vansteenkiste et al., 2007), proactive work behavior (Bilal et al., 2021) commitment (Olafsen et al., 2017; Becker et al., 2018), innovative work behavior (Wang et al., 2021), talent retention (Fowler, 2014; Bock, 2015), and financial performance (Deci et al., 2017), among other necessary performance measures. Finally, SDT narrates a comprehensive model for assessment and suggests a well-defined framework for taking the initiative to enhance and maintain involvement and motivation (e.g., Deci et al., 1989; Hardré and Reeve, 2009).

Entrepreneurial leadership stimulates self-initiated behavior in volatile and uncertain situations and helps attain organizational objectives relating to recognizing and improving entrepreneurial opportunities (Surie and Ashley, 2008). Entrepreneurial leadership stimulates self-initiated behavior in precarious and uncertain situations and helps attain organizational objectives relating to recognizing and improving entrepreneurial opportunities (Surie and Ashley, 2008). Numerous researchers have studied entrepreneurial leadership and have encouraged the bourgeoning of practices described in entrepreneurial leadership (Renko et al., 2015; Bagheri and Akbari, 2018; Cai et al., 2019). This study responds to the call for researchers to investigate the drivers and the mechanism of how proactive work behavior can be induced in the employees (Smithikrai and Suwannadet, 2018). Furthermore, this study adds to the literature on entrepreneurial leadership and its impact on proactive work behavior as well as the psychological wellbeing of employees through the dual mediating mechanism of sustainable employability and work uncertainty.

### Literature Review

To flourish and survive today, a firm must develop competitive strategies to respond to environmental challenges (Pingel et al., 2019) created due to COVID-19. Concerning this epidemic of COVID-19, proactive individuals anticipating changes, instigating advancements, and being prepared to take liability might provide an extensive edge over other firms. Therefore, in organizational behavior studies, proactivity is considered an exclusive anticipated behavior for a long time. Moreover, the prevailing view in the scholarly work is that proactive work behavior leads to accelerated career success and more fantastic results (Thomas et al., 2010; Tornau and Frese, 2013). Proactive behavior is future-oriented, self-initiated behavior to replace and revamp oneself (Parker et al., 2006) and has been established to provide varied work consequences (Bindl and Parker, 2010). Regardless of its advantages, proactive behavior is not certainly easy to stimulate. Proactivity instigates pursuing a revolutionary prospect, which brings uncertainty, meaning the consequence of individual behavior is unknown (Wu and Parker, 2017). Proactivity also includes initiating revolution, which is rarely hailed by employees or managers who mostly favor no change (Morrison and Phelps, 1999; Parker et al., 2010). Individual and entrepreneurial leaders are involved in proactive behavior because this behavior's possible risks and uncertainties offer a helpful situation. Moreover, individuals are stimulated to use substitute means to get their job done by not bothering about latent hurdles, likely accelerating proactivity (Parker et al., 2010).

Entrepreneurial leadership is founded on multicultural leadership views, specifically team-oriented, value-based, and neo-charismatic leadership (Gupta et al., 2004). Some characteristics of entrepreneurial leadership have a close resemblance with transformational leadership, like intellectual stimulation; however, entrepreneurial leaders have a distinction from transformational leaders in some areas of motivation, such as charismatic role modeling and inspirational motivation (Renko et al., 2015). Additionally, transformational leaders practice impression management and charisma to instigate their associates. Additionally, entrepreneurial leaders behave as role models by executing entrepreneurial practices (Renko et al., 2015; Harrison et al., 2018). Finally, the prominent element of transformational leadership is individual consideration; transformational leaders recognize their subordinates' individual needs and skills and maintain a stable communication interface and consider their valuable capabilities (Avolio and Bass, 1995). However, personal consideration is not the focal point of entrepreneurial leaders, but they believe their subordinates are given autonomy and relatedness (Renko et al., 2015). Entrepreneurial leaders boost the employees' self-assurance in entrepreneurial abilities and cultivate an appetite for innovation and creativity (Chen, 2007; Cardon et al., 2009). Therefore, the concept of entrepreneurial leadership is based on opportunity exploration and exploitation activities and their subordinates (Renko et al., 2015).

### Entrepreneurial Leadership and Employee Proactive Behavior

Proactive behavior encompasses discovering an innovative solution to challenge the status quo (Amabile, 1988). Proactive behavior is a significant factor in the long-term existence and success of an organization. Under this behavior, individuals create innovative ideas for modifying and creating new procedures, services, and products. As market demands are progressively becoming more volatile, impulsive, and inconstant, it becomes complex for the leaders to manage each change independently (Owens and Hekman, 2012). Considering this, it is not only the responsibility of the R&D department to introduce creativity and innovation, but now this responsibility has been extended directly or indirectly to all individual levels of the business firm (Bruns and Stalker, 1961; Bai et al., 2016) through the proactive behavior of employees. A leader has been considered an essential impetus in involving employees in self-initiated behavior that may lead to creative behavior (Shalley and Gilson, 2004; Hemlin and Olsson, 2011; Zhou and Hoever, 2014). Previous studies have established that leaders develop creativity (Qu et al., 2015; Chen and Hou, 2016; Koh et al., 2019; Ribeiro et al., 2020) through self-initiated behavior.

In a competitive business environment, entrepreneurial leadership is an encouraging and stimulating factor for the employees that enhances the innovative (Bagheri and Akbari, 2018; Cai et al., 2019) and employees' proactive behavior. Entrepreneurial leaders can efficiently achieve the self-initiative and creative process by encouraging their employees to generate and realize new ideas in a challenging business scenario (Currie et al., 2008). Entrepreneurial leaders act as a role model for subordinates (Renko, 2017). They encourage them to embody the eagerness to behave proactively and involve themselves in innovation (Newman et al., 2018). The entrepreneurial leader has functional capabilities and can encourage their team members to relinquish the traditional way of executing the task and stimulate them to be proactive and invest their energies in innovating something new (Gupta et al., 2004) through proactive behavior. Furthermore, entrepreneurial leaders intentionally empower their subordinates to regulate and inspire them to proactively induce innovation (Surie and Ashley, 2008; Renko et al., 2015).

Entrepreneurial leaders promote autonomy, trust, competence, and relatedness among their team members and act as a role model (Renko et al., 2015). SDT reinforces this by insisting that individuals learn from their mentors and behave accordingly if relatedness and autonomy are on their surge. An entrepreneurial leader's prime duty is to instruct and lead their employees as they should engage in self-initiative behavior (Renko et al., 2015; Harrison et al., 2018). SDT demonstrates that individual autonomy and relatedness obtained from their role models, instructors, and mentors help them handle complex tasks where mistakes are expensive and risky. In such examples, individuals gain competence from a competent leader who knows how to execute such functions without making unwanted errors. Similarly, individuals can implement what they have learned from this autonomy and competence to act proactively on complex tasks. Consequently, based on SDT and the abovementioned discussions, the following hypothesis is proposed:

*Hypothesis 1: Entrepreneurial leadership is directly and positively related to the PWB of employees*.

### Entrepreneurial Leadership and Psychological Wellbeing of Employees

Psychological wellbeing at the workplace is linked with inherent conditions of pleasure observed by an employee, leading to cheerfulness, life satisfaction, and confidence (Massé et al., 1998; Diener et al., 2003). In addition, it emphasizes pleasant cognitive and affective practices (Massé et al., 1998; Diener et al., 2003). Studies proved that workplace psychological wellbeing creates benefits in the best interest of individuals and the organization (Judge et al., 2001; Harter et al., 2002; Lyubomirsky et al., 2005). Employees possess superior psychological wellbeing levels at the individual level, have better immune systems, more energy, and more major social networks (Lyubomirsky et al., 2005). Resultantly, at a firm level, productivity (Harter et al., 2002), individual performance (Judge et al., 2001), quality of work, cooperation, and creativity (Lyubomirsky et al., 2005) are improved.

SDT emphasizes that individuals having primary desires for *relatedness, competence, and autonomy* are imperative throughout life in all humanities (Deci and Ryan, 2008, p. 182). Fulfilling these needs stimulates an individual's innate inclination toward psychological wellbeing and growth, while dissatisfaction with such necessities leads to ill-being and psychopathology (Ryan et al., 2006). The need for *competence* is related to the sense of self-efficaciousness, and the need for *relatedness is associated with the perception that individuals feel linked and favored by others* (Deci and Ryan, 2008, p. 183). The need for autonomy is the perception that a particular action is voluntary and self-imposed. Experience of entrepreneurship in the workplace influences the psychological states of employees (Parasuraman et al., 1996). Entrepreneurship is an optimistic mind-state when the track to attaining an objective is unambiguous for subordinates to have the autonomy to achieve that objective. In response to that, employees observe more confidence in achieving their goals. An optimistic state of mind leads to improving the psychological wellbeing of an employee. Achieving own individual goals is a sign of an individual's subjective wellbeing (Brunstein, 1993; McGregor and Little, 1998).

Self-determination is a critical facet of entrepreneurship, which is demonstrated as intrinsic motivation with the following features: autonomy, relatedness, and competence, displaying an individual's attitude toward their task role. Entrepreneurship can also affect other job-associated parts of psychological wellbeing, such as workload and skill exploitation. Therefore, an entrepreneurial leader may be an active resource to obtain satisfaction at work and reduce job stress when the subordinates' amount of work is amplified. Entrepreneurial leadership emphasizes the positive relationship between leader and subordinate and manages the individual's psychological problems to enhance motivation. Moreover, having a sense of entrepreneurship minimizes psychological distress. Based on the above debate, the following hypothesis is proposed:

*Hypothesis 2: Entrepreneurial leadership is directly and positively related to the wellbeing of employees*.

### Sustainable Employability and PWB

With the decline of the young expert workforce, sustainable employability (SE) is an emerging area of interest for all humanities and numerous employees depart the industry for health problems (Van den Heuvel, 2013; De Jonge and Peeters, 2019). Sustainable employability is referred to as the prospect for employees to “make a valuable contribution through their work, now and in the future, while safeguarding their health and welfare” (Pejtersen et al., 2010; Van der Klink et al., 2016). Sustainable personnel is essential for an organization to diminish the expenses of absenteeism and turnover due to workers' lousy health and non-conducive work environments that ultimately lead to condensed productivity (Shikiar et al., 2001; Roczniewska et al., 2020). Therefore, sustainable employability is imperative, particularly in SMEs. As it helps retain experts whose substitutes are challenging because of their expertise, skills, and education. It has been proven that the expense of training and developing newcomers is higher than retaining the current employees (Emami et al., 2012). The study justified that the ordinary expense of an employee turnover is 150% of the individual's salary (Ramlall, 2003). Zinser (2003) describes how employability features comprise a range of work-related skills and personality attributes, namely, proactive behavior, personality preferences, an upper level of self-confidence and self-esteem, and emotional intelligence.

A comfortable and healthy working life is essential for maintaining or stimulating adaptability, motivation, and proactive behavior. These are critical features of sustainable employability (Van der Klink et al., 2016). Employees possessing sustainable employability are enthusiastically stimulated in handling and harmonizing their job to their values and competencies (SER, 2009; Van der Klink et al., 2016) and actively involved in self-initiative behavior such as proactive behavior.

*Hypothesis 3: Sustainable employability is directly and positively related to PWB*.

### Sustainable Employability and Wellbeing

Strategically, knowledge is an essential resource for an organization (Grant, 1996); it encompasses the knowledge in employees' minds that occasionally cannot be transmuted to explicit knowledge and is reserved until the employee is employed there. Consequently, the removal of the workforce could result in the loss of knowledge and vital skills. Furthermore, an elevated level of turnover carries adverse effects; for instance, it may lead to team instability, an amplified workload on the remaining team members, and poor work performance. Therefore, a healthy and stable workforce is essential for SMEs. Sustainable employability is that workers can remain functional for the whole span of their lives and retain their fitness and wellbeing (Van der Klink et al., 2016). Second, sustainable employability is concerned with employees' productivity and comprises good physical and psychological health and wellbeing at the workplace.

Wellbeing can be defined as a process that gives a sense of purpose and satisfies an employee's needs related to a personal relationship, financial and without financial rewards, and attractive environments (La Placa et al., 2013). Positive psychological wellbeing generally comprises six characteristics, namely, personal optimization, life satisfaction, positive emotion, prosocial behavior, multiple dimensions, dynamic recreation of wellbeing, and equilibrium of attributes. The psychological wellbeing of employees can enhance the sustainability of the firm. However, wellbeing is not as simple as getting happiness, but the motivation for excellence signifies recognizing an individual's true potential (Ryff and Keyes, 1995). According to self-determination theory, sustainable employability is stimulated by a diversity of impetuses that fluctuate along with a range of autonomy (Deci and Ryan, 1985; Ryan and Deci, 2017). Intrinsic motivation (behaving for inner satisfaction) is the supreme autonomous practice of motivation. Conversely, when sustainability is not intrinsically pleasant, the individual may be autonomously stimulated through integrated regulation (behaving according to their objectives and ethics) and recognized principles (acting to achieve individually valued consequences). Durable sustainability is impossible when action is not autonomous but enforced by some external drive (e.g., Ng and Kee, 2012).

Due to autonomous motivation, involvement in positive behavior leads to more adaptive health upshots, comprising better behavioral implementation, maintenance, and more positive wellbeing (Deci and Ryan, 2008). Further autonomous inspiration is assisted through the gratification of three basic psychological needs, namely, relatedness, competence, and autonomy (Ryan and Deci, 2000, p. 69). Wellbeing is a six-dimensional process with distinctive features such as positive associations with others, environmental mastery, objectives in life, self-acceptance, individual growth, and autonomy. Currently, employees spend most of their time at the workplace due to high work demand and sustained work pressure. Consequently, providing a hygienic and sustainable workplace with better employee-focus HR practices is anticipated to assist employees in improving productivity and creating a satisfactory employee-centric climate that leads to employees' superior wellbeing. Furthermore, sustainable employability is supposed to give a tremendous competitive advantage over rival firms (Ghoshal et al., 1997) and might improve the employees' wellbeing.

*Hypothesis 4: Sustainable employability is directly and positively related to wellbeing of employees*.

### Work Uncertainty and Proactive Work Behavior

Proactive behavior is self-initiated, future-oriented behavior having the objectives of improving the situation or oneself (Parker and Collins, 2010, p. 635). Work uncertainty occurs in an organization where tasks' inputs, processes, or outputs have no likelihood (Wall et al., 2002). The uncertainty factors include evolving market demands, technological modification, and high competition (Bruns and Stalker, 1961). The degree of uncertainty in a firm defines which specific work behavior should be used, whether to formalize behavior rather than emergent behavior like proactive or adaptive behavior. In emergent behavior, the degree of uncertainty is very high. In SMEs, the resources are limited (Woschke et al., 2017); leaders and their subordinates are not ready to take a risk. On the contrary, jobs are at stake in uncertain conditions by being proactive, and in this way, proactive behavior involves uncertainty.

*Hypothesis 5: Work uncertainty is negatively related to PWB*.

### Work Uncertainty and Psychological Wellbeing

Self-determination theory postulates that individuals involved in activities seek valuable, significant motivation (Ryan and Deci, 2000). A job-related task gives relational, social, and psychological gratification. Work tasks improve wellbeing by letting an individual fulfill their needs for belongingness, affiliation, and survival (Abildgaard et al., 2020). Observing themselves, acquiring new knowledge and skills, achieving different tasks, and sharing cognitive and emotional support with colleagues enhance self-confidence and self-worth (Obrenovic et al., 2015). Moreover, one of the basic human needs is the desire to relate, stay attached or linked, and permit effective work and individual growth (Flum, 2015). On the contrary, work uncertainty like fear of losing a job and dipping into financial crisis leads to loneliness, cognitive dissonance, identity disturbance, and ultimately, mental sickness (Blustein et al., 2020).

*Hypothesis 6: Work uncertainty is negatively related to employee wellbeing*.

### Work Uncertainty and Sustainable Employability as Mediators

**Figure 1:**
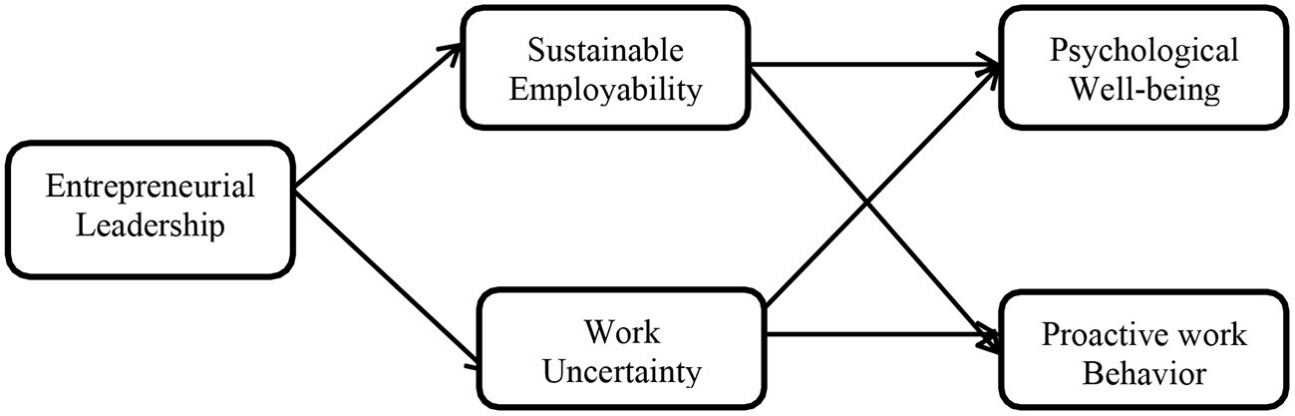
Conceptual model.

The hypotheses are entrenched in the framework in Figure 1, and the model instituted a concurrent investigation of the hypotheses, especially key to the study's model is the process of mediation. We suggest that entrepreneurial leadership's motives are associated with employees' proactive work behavior and psychological wellbeing. Furthermore, employees' perceptions of work uncertainty and sustainable employability mediate the associations of entrepreneurial leadership with employees' proactive work behavior and psychological wellbeing. Thus, entrepreneurial leadership is connected to the two criterion variables that may impact employee perceptions.

*Hypothesis 7: Employees' work uncertainty and sustainable employability mediate the relationships between entrepreneurial leadership with proactive work behaviors and psychological wellbeing*.

## Methodology

The data were collected from SMEs related to construction, publishing, pharmaceutical, printing, and IT sectors employees. The data were collected in three waves, as Podsakoff et al. (2012) recommended that the predictor and criterion variables may be measured separately to reduce the common method bias. The obtained data were collected in three waves. In the first wave, T1 was circulated to get data related to entrepreneurial leadership and demographic variables. Then, second-wave data were collected related to mediating variables like sustainable employability and work uncertainty. Finally, third-wave data was collected related to psychological wellbeing and proactive work behavior. There was a 3-week gap in each wave as its suitable and the same interval was applied in the previous leadership studies (e.g., Neubert et al., 2008; Zohar and Polachek, 2017; Demirtas, 2015; Kim and Beehr, 2017). Proactive work behavior data were collected from employees' respective supervisors to reduce the common method bias further. Initially, 350 survey forms were circulated to collect data; 314 were received in the first wave, 269 were received in the second wave, and 236 were received in the last third wave. A total of 218 survey forms were considered for statistical data analysis as 18 survey forms were removed due to incomplete data. Out of 218 participants, 135 were male, and 83 were female.

### Measures

This study used already established instruments that have been extensively used and validated in the existing literature.

#### Entrepreneurial Leadership (Independent Variable)

An eight-item scale developed by Renko et al. (2015) was used to measure entrepreneurial leadership. Responses were calculated on a five-point Likert scale ranging from 5 strongly agree to 1 strongly disagree. Sample items were “My team leader has creative solutions to problems” and “My team leader demonstrates a passion for his/her work.” Cronbach's α value was 0.70.

#### Sustainable Employability (Mediator)

Sustainable employability was measured using job satisfaction, health, and job performance as measured in previous studies (Roczniewska et al., 2020). To measure job satisfaction, applied *three* items were developed by Hellgren et al. (1997). This scale measures the extent of happiness and joy with one's work (an example item is “I enjoy my work”). Next, health was measured with one item from the COPSOQ II (2014). “In general, I would say my health is good.” with answers ranging from (1) entirely disagree to (5) fully agree. Finally, job performance was measured using three items from a workplace productivity scale (Bindl and Parker, 2011). The scale consisted of items concerning the efficiency, the quality, and the amount of work (e.g., “How would you describe your efficiency at work in the last week?”), and its response was rated on a 5-point Likert scale from 1 (*low*) to 5 (*high*). The same scales were used in previous research to measure sustainable employability (Roczniewska et al., 2020). All three dimensions of sustainable employability (i.e., job satisfaction, health, and job performance) were taken as composite variables in this study. The composite reliability of sustainable employability was 0.76.

#### Work Uncertainty (Mediator)

Work uncertainty was measured using a 9-item scale developed by Leach et al. (2013). Sample items are “Does the equipment you use work reliably?” and “Do you come across unexpected problems in your work?” Responses were recorded on a 5-point response scale from “Not at all” (1) to “A great deal” (5). Cronbach's α value was 0.74.

#### Proactive Work Behavior (Dependent Variable)

Proactive work behavior was assessed with a 13-item scale of Parker and Collins (2010). Sample items are “How frequently do you promote and champion ideas to others?” and “How frequently do you try to institute new work methods that are more effective?” Responses will be rated on a 5-point Likert scale (ranging from 5 = very frequently, 1 = very rarely). Cronbach's α value was α 0.79.

#### Psychological Wellbeing (Dependent Variable)

A five-item scale of Heun et al. (2001) was utilized to determine the psychological wellbeing of employees. Sample items are “I felt calm and relaxed” and “I felt active and vigorous.” Responses were made on a scale ranging from 1 = strongly disagree to 5 = strongly agree. Cronbach's α value was 0.75.

The predictor variable entrepreneurial leadership in the model was negatively correlated to work uncertainty (*r* = −0.48, *p* < 0.01; Table 1) and positively related to sustainable employability (*r* = 0.64, *p* < 0.01); the entrepreneurial leadership was also correlated to the criteria (proactive work behavior, *r* = 0.53, *p* < 0.01, and psychological wellbeing, *r* = 0.57, *p* < 0.01). The mediator work uncertainty was negatively related to the proactive work behavior (*r* = −0.40, *p* < 0.01) and psychological wellbeing (*r* = −0.61, *p* < 0.01); sustainable employability was related to proactive work behavior (*r* = 0.50, *p* < 0.01) and psychological wellbeing (*r* = 0.35, *p* < 0.01).

**Table 1:** Descriptive statistics and correlations (*N* = 218).

**Variables**	**Mean**	**SD**	**1**	**2**	**3**	**4**	**5**	**6**	**7**	**8**	**9**
1. Gen.	0.62	0.49									
2. Age	2.15	0.89	0.04								
3. Edu.	2.43	0.63	−0.13	0.08							
4. Exp.	2.12	0.91	0.04	0.96^**^	0.06						
5. WU	1.45	0.46	−0.04	0.10	0.06	0.08	**(0.74)**				
6. SE	4.14	0.63	0.03	−0.09	0.01	−0.11	−0.24^**^	**(0.76)**			
7. PWB	4.07	0.48	0.01	−0.02	−0.02	−0.03	−0.40^**^	0.50^**^	**(0.79)**		
8. WB	4.47	0.58	0.09	−0.02	−0.12	−0.03	−0.61^**^	0.35^**^	0.45^**^	**(0.75)**	
9. EL	4.40	0.46	0.04	−0.01	−0.06	−0.01	−0.48^**^	0.64^**^	0.57^**^	0.53^**^	**(0.70)**

** *Correlation is significant at the 0.01 level (2-tailed)*.

*Gen, gender; Edu., education; Exp., experience; WU, work uncertainty; SE, sustainable employability; PWB, proactive work behavior; WB, Psychological wellbeing; EL, entrepreneurial leadership*.

*Reliability value of study variables are presented in parenthesis in bold letters*.

### Hypotheses and Model Testing

The statistical hierarchal regression analysis resulted presented in Table 2 significantly proved hypothesis 1 (ΔR^2^ = 0.33, β = 0.059, *p* < 0.000, model 3) and hypothesis 2 (ΔR^2^ = 0.27, β = 0.65, *p* < 0.000, model 8) that entrepreneurial leadership is positively related with proactive work behavior and psychological wellbeing of employees respectively. Sustainable employability has significant positive relationship with proactive work behavior (ΔR^2^ = 0.25, β = 0.39, *p* < 0.000, model 4) and psychological wellbeing (ΔR^2^ = 0.12, β = 0.32, *p* < 0.000, model 9). These results statistically proved hypotheses 3 and 4 that sustainable employability has a positive relationship with proactive work behavior and psychological wellbeing. Similarly, hypotheses 5 and 6 were also statistically significant as there is a negative relationship between work uncertainty and proactive (ΔR^2^ = 0.16, β = −0.42, *p* < 0.000, model 5) and psychological wellbeing (ΔR^2^ = 0.37, β = −0.77, *p* < 0.000, model 10).

**Table 2:** Hierarchical regression results.

	**SE**	**WU**	**PWB**				
**Variables**	**Model**	**Model**	**Model**	**Model**	**Model**	**Model**	**Model**
	**1**	**2**	**3**	**4**	**5**	**6**	**7**
Gender	0.02	−0.02	−0.01	−0.01	−0.01	−0.02	−0.02
Age	0.13	0.137	0.06	−0.01	0.10	0.03	0.08
Education	0.05	0.02	0.01	−0.02	–	–	0.01
Tenure EL	−0.2	−0.09	0.06	0.02	−0.09	−0.03	−0.09
SE	0.87^***^	−0.48^***^	0.59^***^	0.39^***^		0.44^***^	0.51^***^
WU					−0.42^***^	0.18^***^	−0.18^**^
Adj. R^2^	0.41	0.23	0.31	0.23	0.15	0.34	0.33
F Value	31.35^***^	13.64^***^	20.54^***^	14.29^***^	8.37^***^	19.49^***^	18.83^***^
ΔR^2^	0.41	0.23	0.33	0.25	0.16	0.36	0.35

* *p < 0.05,*

** *p < 0.01,*

*** *p < 0.001.*

According to Baron and Kenny (1986), a variable works as a mediator when it fulfills the following three conditions: (1) Variations in the levels of the predictor variable (entrepreneurial leadership) significantly account for variations in the supposed mediator (sustainable employability and work uncertainty). (2) Variations in the mediator (sustainable employability and work uncertainty) significantly account for variations in the criterion variable (psychological well- being and proactive work behavior). (3) When the independent and mediating variables are controlled, a significant prior association between the predictor and criterion variables is no longer significant or reduced in their significant values. Condition one was checked with a regression where, in the first step, the demographic variables were introduced, and in the second step, the entrepreneurial leadership was presented. The model 1 (Table 2) predicts that entrepreneurial leadership has positive relationship with sustainable employability (Δ*R*^2^ = 0.41, β = 0.87, *p* < 0.000, model 1) controlled by demographic variables shown in Table 2. Condition two was checked in model 6 that sustainable employability has significantly (Δ*R*^2^ = 0.36, β = 0.18, *p* < 0.01) positive relationship with proactive work behavior.

Condition three was analyzed with a regression where entrepreneurial leadership and sustainable employability were inducted as predictor variables and proactive work behavior as a criterion variable, controlled by demographic variables. The model 6 shows a significant β value (0.44, *p* < 0.000) of entrepreneurial leadership, but its value was reduced from 0.59 to 0.44. These results proved that sustainable employability partially mediates the relationship between entrepreneurial leadership and proactive work behavior. Result of model 8 proved that entrepreneurial leadership has positive relationship with psychological wellbeing of employees (Δ*R*^2^ = 0.27, β = 0.65, *p* < 0.000). Condition two was evaluated in model 11 (Table 3) and found sustainable employability has no significant relationship with psychological wellbeing (Δ*R*^2^ = 0.27, β = 0.0.02, *p* < 0.01). These results showed that sustainable employability did not mediate the relationship between entrepreneurial leadership and psychological wellbeing.

**Table 3:** Hierarchical regression results.

	**WB**				
**Variables**	**Model**	**Model**	**Model**	**Model**	**Model**
	**8**	**9**	**10**	**11**	**12**
Gender	0.07	0.08	0.07	0.07	0.06
Age	0.09	0.04	0.19	0.09	0.18
Education	−0.07	−0.11	−0.07	0.08	−0.06
Tenure	−0.11	−0.03	−0.17	−0.1	−0.16
EL	0.65^***^			0.63^***^	0.37^***^
SE		0.32^***^		0.02	
WU			−0.77^***^		−0.59^***^
Adj. *R^2^*	0.27	0.12	0.38	0.27	0.44
*F*-value	17.35^***^	7.06^***^	27.47^***^	14.41^***^	29.91^***^
Δ*R*^2^	0.27	0.12	0.37	0.27	0.44

*SE, sustainable employability; WU, work uncertainty; PWB, Proactive work behavior; WB, Psychological wellbeing*.

^*^p < 0.05,

^**^p < 0.01,

*** *p < 0.001*.

The model 2 predicts that entrepreneurial leadership has negative relationship with work uncertainty (Δ*R*^2^ = 0.23, β = −0.48, *p* < 0.000, model 2) controlled by demographic variables shown in Table 2. Furthermore, for work uncertainty, condition two was assessed in model 7 and found work uncertainty has a significant negative relationship with proactive work behavior (Δ*R*^2^ = 0.35, β = −0.18, *p* < 0.01). Condition three was analyzed with a regression where entrepreneurial leadership and work uncertainty were inducted as predictor variables and proactive work behavior as a criterion variable, controlled by demographic variables. The model 7 shows a significant β value (0.51, *p* < 0.01) of entrepreneurial leadership but its beta value was reduced from 0.59 to 0.51. These results proved that work uncertainty partially mediates the relationship between entrepreneurial leadership and the proactive work behavior of employees. Finally, to confirm the mediation effect of work uncertainty with entrepreneurial leadership and the psychological wellbeing of employees, model 12 (Table 3) showed that work uncertainty has a significant positive relationship with the psychological wellbeing of employees (Δ*R*^2^ = 0.44, β = −0.59, *p* < 0.000).

Condition three was investigated with a regression where entrepreneurial leadership and work uncertainty were inducted as predictor variables and psychological wellbeing as a criterion variable, controlled by demographic variables. The model 12 results showed a significant β value (0.37, *p* < 0.01) of entrepreneurial leadership and its β values were reduced from 0.65 to 0.37. These results proved that work uncertainty partially mediates the relationship between entrepreneurial leadership and the psychological wellbeing of employees.

### Mediation and Bootstrapping

Moreover, to calculate bootstrapping results for meditation analysis as a computational instrument to confirm the mediation further, Hayes and Scharkow's (2013) PROCESS macro was performed. Table 4 offers the direct effects and bootstrapped approximations with 95% confidence intervals to gain indirect effects. K^2^ (kappa squared) mediation effect size is also stated by following the recommendations of Preacher and Kelley (2011). *k*^2^ is the ratio of the indirect effect to the maximum possible extent of the indirect impact given the constraints of the data (Hayes and Scharkow, 2013) is not sensitive to sample size. The principles of Cohen's guidelines defining small (0.01), medium (0.09), and large (0.25) effect sizes were considered (Preacher and Kelley, 2011) to describe the magnitude of effect sizes. Sustainable employability and work uncertainty mediated the relationships between the two outcomes, namely, psychological wellbeing and proactive work behavior, and entrepreneurial leadership, as their confidence intervals did not include zero, supporting mediation effects except for psychological wellbeing, which was not mediated by sustainable employability as there was zero value between the upper and lower limit of confidence interval (−0.13, 0.18). For instance, the relationship between entrepreneurial leadership as a forecaster and proactive work behavior as an outcome was mediated by sustainable employability. As shown in the first row of Table 4, the direct effect of entrepreneurial headship on proactive work behavior was significant (*c*′ = 0.59, *p* = 0.000). The indirect impact of entrepreneurial leadership on proactive work behavior *via* sustainable employability was noteworthy (*ab* = 0.15, confidence interval [95%]: lower limit = 0.04, upper limit = 0.27, *k*^2^ = 0.14), and the effect size of *k*^2^ can be read as a medium concerning Cohen's standard. These results significantly proved that sustainable employability mediates the relationship between entrepreneurial leadership and proactive work behavior.

**Table 4:** Results of bootstrapping tests for estimating indirect effects with 95% confidence intervals (CIs).

			**Direct effect**			**Indirect effects**		
**Predictor**	**Mediator**	**Outcome**	**β (SE)**	**ab**	**SE**	**95% CI**	**abcs**	***k* ^2^**
EL	SE	PWB	0.59*** (0.07)	0.15	0.06	[0.04, 0.27]	0.15	0.14
		WB	0.64*** (0.09)	0.02	0.08	[−0.13, 0.18]	0.02	0.02
		PWB	0.64*** (0.09)	0.08	0.04	[0.03, 0.32]	0.08	0.09
		WB	0.38*** (0.05)	0.28	0.05	[0.33, 0.66]	0.23	0.23

*N = 218. β = c′ (direct effect). SE, bootstrap standard error; ab, unstandardized indirect effect. 95% CI, SE, and ab were obtained from 10,000 bootstrap samples. k2, indirect effect/maximum possible mediation; abcs, completely standardized indirect effect*.

However, the case with other models in the second row of Table 4, the direct effect of entrepreneurial leadership to employees' wellbeing was significant (*c*′ = 0.64, *p* = 0.000), and the indirect effect of entrepreneurial leadership on psychological wellbeing through sustainable employability was not significant (*ab* = 0.02, CI [95%]: LLCI = −0 p. 13, ULCI = 0.18, *k*^2^ = 0.02). Thus, these results statistically confirmed that sustainable employability did not mediates the relationship between employees' entrepreneurial leadership and psychological wellbeing. The result as shown in row three of Table 4, significantly proved the mediation effect (*c*′ = −0.51, *p* = 0.000; *ab* = 0.08, CI [95%]: LLCI = 0.03, ULCI = 0.32, *k*^2^ = 0.09) of work uncertainty between entrepreneurial leadership and proactive work behavior. Work uncertainty mediates the relationship between entrepreneurial leadership and psychological wellbeing of employees as presented in row four of Table 4 (*c*′ = 0.38, *p* = 0.000; *ab* = 0.28, CI [95%]: LLCI = 0.33, ULCI = 0.66, *k*^2^ = 0.23).

## Discussion

The impetus of this study was to describe the effects of entrepreneurial leadership on employees' proactive behavior and psychological wellbeing by investigating the prospective arbitrating role of sustainable employability and work uncertainty. Thus, elucidating the mechanisms by which entrepreneurial leadership influences employees' proactive behavior and wellbeing. The results generally supported the theorized relationships. Entrepreneurial leadership may affect employee behaviors to the point that it tips toward sustainable employability and work uncertainty. Entrepreneurial leaders inspire initiative and responsibility toward the work proficiency of employees, improve spirits of sustainable employability (including health, satisfaction, and job performance), reduce the perception of work uncertainty, and are involved in proactive work behaviors, and enhance employees' psychological wellbeing.

In addition to improving proactive work behavior, work uncertainty also enhances the psychological wellbeing of employees, although sustainable employability may not impact the psychological wellbeing of employees. Followers' performance can be increased if leaders can reduce subordinates' perception of work uncertainty by providing professional challenges and training with superior values, a feature of entrepreneurial leadership. In addition, substantial results on the positive side of leadership with proactive work behavior (Schmitt et al., 2016; Wu and Parker, 2017; Hu et al., 2018) were found to be consistent in the previous studies. Consequently, this study adds to the entrepreneurial leader's literature by explaining how it may affect employees' behavior and wellbeing.

Concerning the particular outcome variables in this study, the previous studies investigating the effect of entrepreneurial leadership on contextual performance with proactive behaviors are limited, while some researchers have proposed positive connections between entrepreneurial leadership and other positive outcomes such as creativity and innovation (Renko et al., 2015; Bagheri, 2017; Bagheri and Akbari, 2018; Cai et al., 2019). Furthermore, contrary to previous studies focusing on positive employee perception, this study investigated the association of entrepreneurial leadership with work uncertainty.

By promoting sustainable employability and inhibiting work uncertainty, entrepreneurial leaders may stimulate employees' good behaviors and psychological wellbeing. However, sustainable employability did not impact employees' psychological wellbeing. Work uncertainty can inhibit organizational effectiveness, sustainable employability, proactive work behavior, and the psychological wellbeing of employees. Moreover, work uncertainty is a significant predictor of organizational performance. Poor organizational belongingness, lousy health, unclear self-identity, and unsatisfied needs may hamper organizational prosperity. Both proactive work behavior and psychological wellbeing are important outcome variables affected by entrepreneurial leadership.

Overall, entrepreneurial leadership can improve the sustainability of employees and reduce work uncertainty in current business organizations. Firms with entrepreneurial leaders possessing the quality of exploration and exploitation instigate subordinates to perceive their behaviors and handle work uncertainties tactfully. Consequently, developing competence, guidance, recognition, motivational support, fostering autonomy, promoting positive relationships, role modeling (entrepreneurial leadership), and motivational support are essential for constructing a sense of sustainable employability and reducing work uncertainty.

Contrary to our prognosis, sustainable employability did not mediate the link between entrepreneurial leadership and psychological wellbeing. One potential explanation for these verdicts may be associated with the construal level of the items for sustainable employability compared to work uncertainty items. In social psychology, the construal level can be defined as the level of conceptualization in which individuals emotionally signify situations, events, or objects (Burgoon et al., 2013). Low construal levels implicate a specific description of problems and things and a total concentration on the “here and now,” in comparison, and high construal levels are inclined to emphasize the conceptual characteristics of situations and objects and focus on theoretical events and the future (Trope and Liberman, 2010). Therefore, sustainable employability items may indicate a high construal level as they stress theoretical detail and the future objectives of the firm. In comparison, work uncertainty items may indicate a low construal level, concentrating on the existing resources that individuals can gain presently.

“Construal level fit” theory can be described in a way that individuals have an inclination for particular things that fit their construal levels (Berson et al., 2015). In our study, the impact of sustainable employability on psychological wellbeing was not significant; maybe the maximum participants of our research had comparatively low construal levels. Unfortunately, we could not investigate this likelihood in this study as we did not assess individual construal levels. Nevertheless, we encourage upcoming researchers to discover how construal levels may affect the psychological wellbeing of employees.

### Limitations and Future Research

Similar to other studies, this research also has some limitations, which opens new avenues for future research. First, this research utilized multisource data to minimize common method variance. Employees' proactive work behavior and entrepreneurial leadership were assessed from a static perspective. Therefore, these assessments were just general evaluations. Entrepreneurial leadership interfaces with employees in a dynamic way (Lingo, 2020), and employees' proactive behaviors are also dynamics (Sylva et al., 2019). Therefore, future studies should investigate the impact of entrepreneurial leadership on proactive employee behavior from a dynamic development perspective. Second, to reduce common method bias, we used a predictive time lag research design as executed by previous researchers (e.g., Neubert et al., 2008; Demirtas, 2015; Kim and Beehr, 2017; Zohar and Polachek, 2017; Bilal et al., 2021). The robust causal implication could not be drawn based on the time lag study. In the future, an experimental research design study should be executed to investigate the causal association between entrepreneurial leadership and proactive work behavior and the psychological wellbeing of employees.

Comparatively, the employees' perceptions about their supervisors' entrepreneurial behaviors are measured by a leadership variable rather than a precise objective measure of the behaviors. Thus, it would be necessary to manipulate the leaders' behavior to operationalize entrepreneurial leadership in an experiment. Besides, experimental manipulations would provide more substantial evidence about the effects of entrepreneurial leaders' behaviors. This study included both proactive behavior and psychological wellbeing; however, to better understand the contribution of entrepreneurial leader behaviors in the workplace, many other potential criteria need to be explored. Other consequences of entrepreneurial leadership, such as positive and negative behavior of employees' work happiness and life satisfaction, are variables for future research. In addition, autonomy provided by entrepreneurial leadership is closely associated with job stressor appraisals, which is likely to impact employees' job satisfaction or turnover intention in return. Finally, future research could also explore boundary conditions that mitigate or accentuate the strength of relations hypothesized in the present study. For example, employees with a strong need for a cooperative team climate may respond more favorably to entrepreneurial leadership, and those with a proactive personality may respond *vice versa*.

## Conclusion

Managers who demonstrate entrepreneurial behavior and flexibility in response to COVID-19 can potentially influence the behaviors of their colleagues. However, managing the practices of entrepreneurial leadership during and after COVID-19 needs proactive planning and renewed attention. Consequently, SME managers need to nurture entrepreneurial behavior and professional flexibility while empowering, motivating, and encouraging their teams to address these challenges.

Conclusively, the integration of self-determination theory improves our comprehension on the effect of entrepreneurial leadership on employee proactive work behavior and psychological wellbeing. This research extends our understanding that work uncertainty and sustainable employability develop a mediating mechanism between entrepreneurial leadership and proactive work behavior and psychological wellbeing. Our study imparts a novel view for contemplating when and how entrepreneurial leadership may expedite employee proactive work behavior and psychological wellbeing. The leaders can exercise our research findings to nurture sustainable employability and reduce work uncertainty in organizations by employee's entrepreneurial behavior for encouraging proactive work behavior and psychological wellbeing.

## Data Availability Statement

The original contributions presented in the study are included in the article/supplementary material, further inquiries can be directed to the corresponding author/s.

## Ethics Statement

Ethical review and approval was not required for the study on human participants in accordance with the local legislation and institutional requirements. Written informed consent for participation was not required for this study in accordance with the national legislation and the institutional requirements.

## Author Contributions

All authors listed have made a substantial, direct, and intellectual contribution to the work and approved it for publication.

## Conflict of Interest

The authors declare that the research was conducted in the absence of any commercial or financial relationships that could be construed as a potential conflict of interest.

## Publisher's Note

All claims expressed in this article are solely those of the authors and do not necessarily represent those of their affiliated organizations, or those of the publisher, the editors and the reviewers. Any product that may be evaluated in this article, or claim that may be made by its manufacturer, is not guaranteed or endorsed by the publisher.
